# Supplementary figures and images for: Establishment and characterization of canine mammary tumoroids for translational research
Source: BMC Biol. 2023 Feb 3;21:23. doi: 10.1186/s12915-023-01516-2 (PMC9898911; doi:10.1186/s12915-023-01516-2)

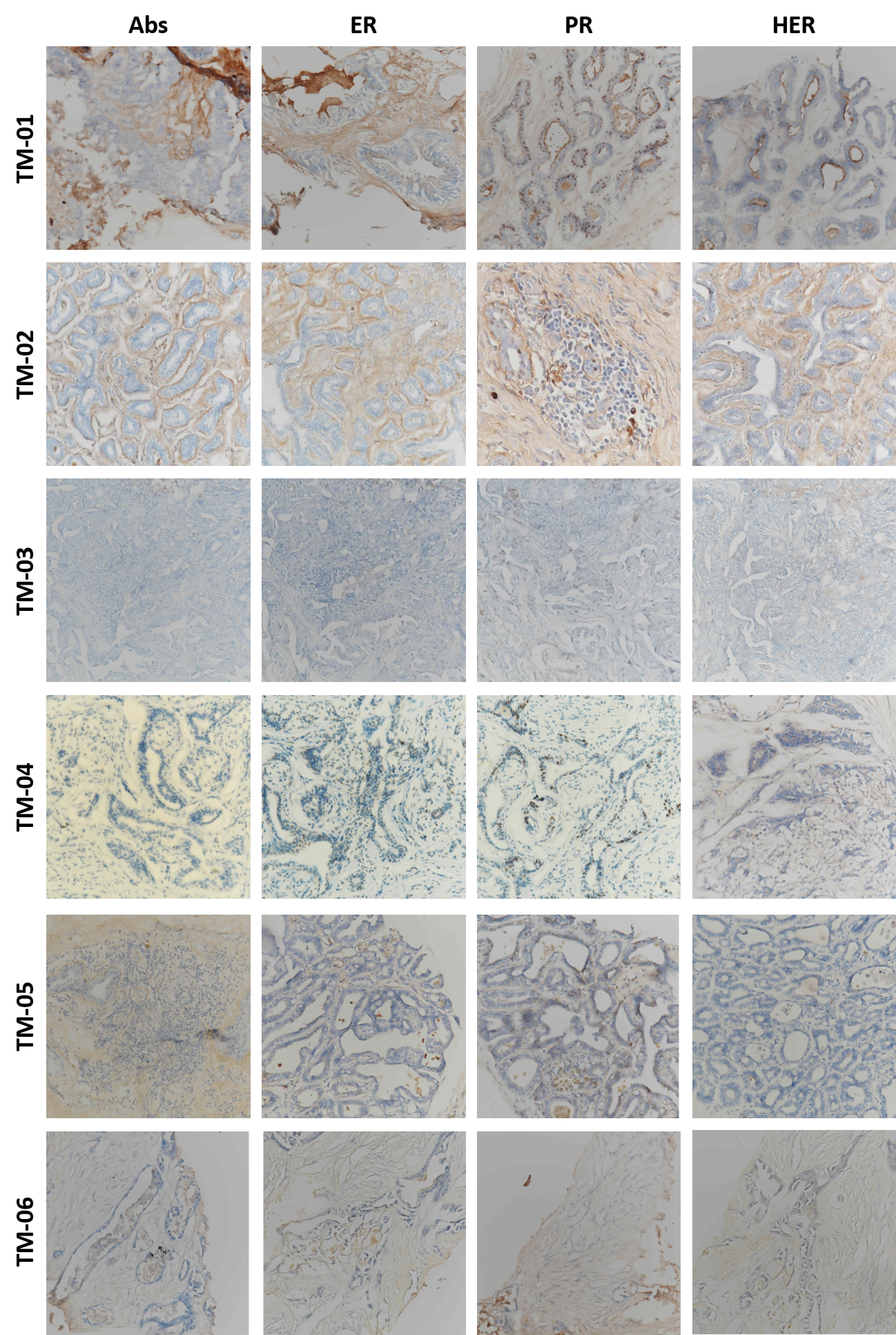

Supplement: Supplementary file 2 — Additional file 2: Table 1. Summary table of canine tumors used in the study. [file 12915_2023_1516_MOESM2_ESM.tif]

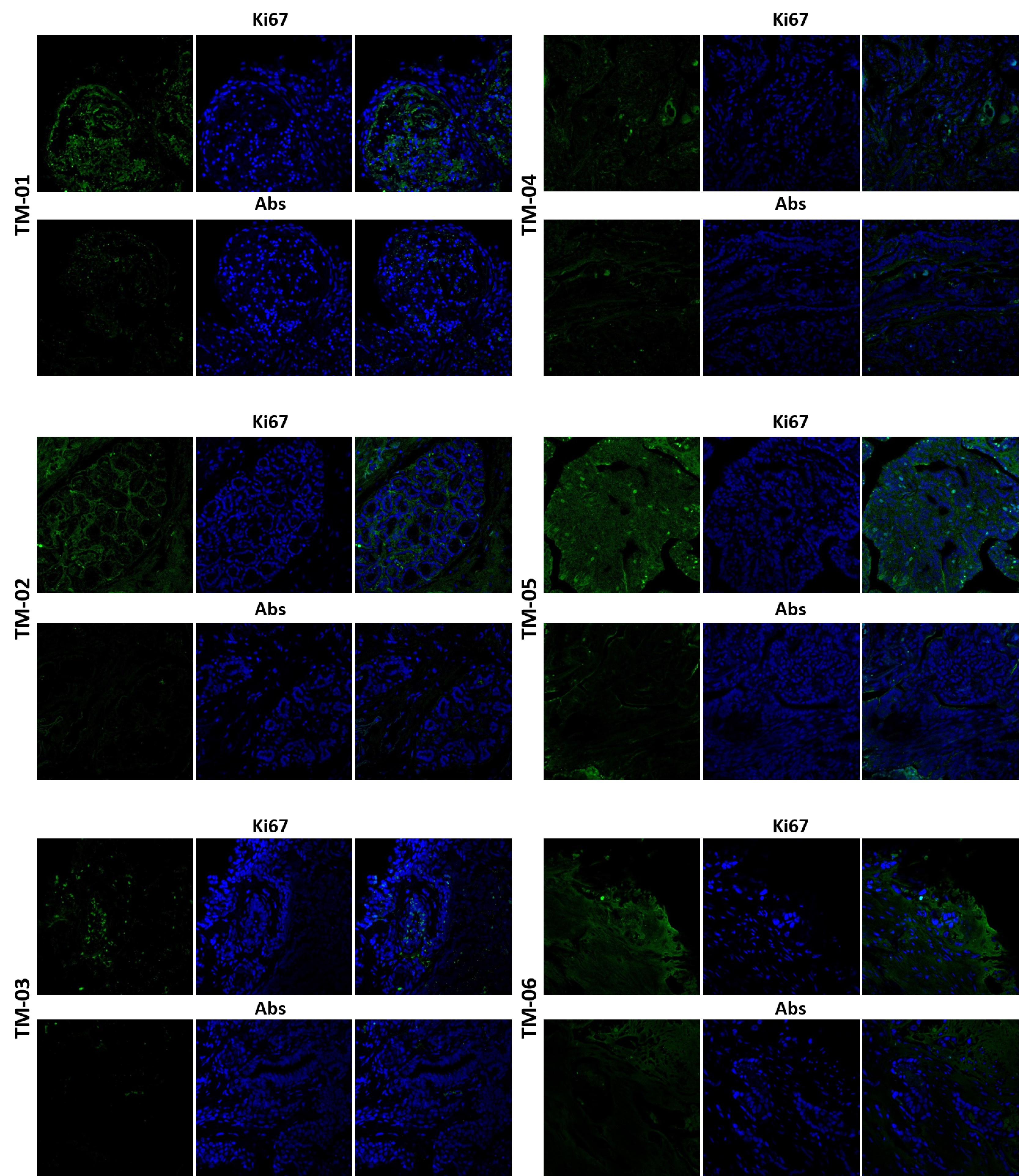

Supplement: Supplementary file 3 — Additional file 3: Fig. S2. Immunofluorescence images of Ki67 stained canine mammary tumors. [file 12915_2023_1516_MOESM3_ESM.tif]

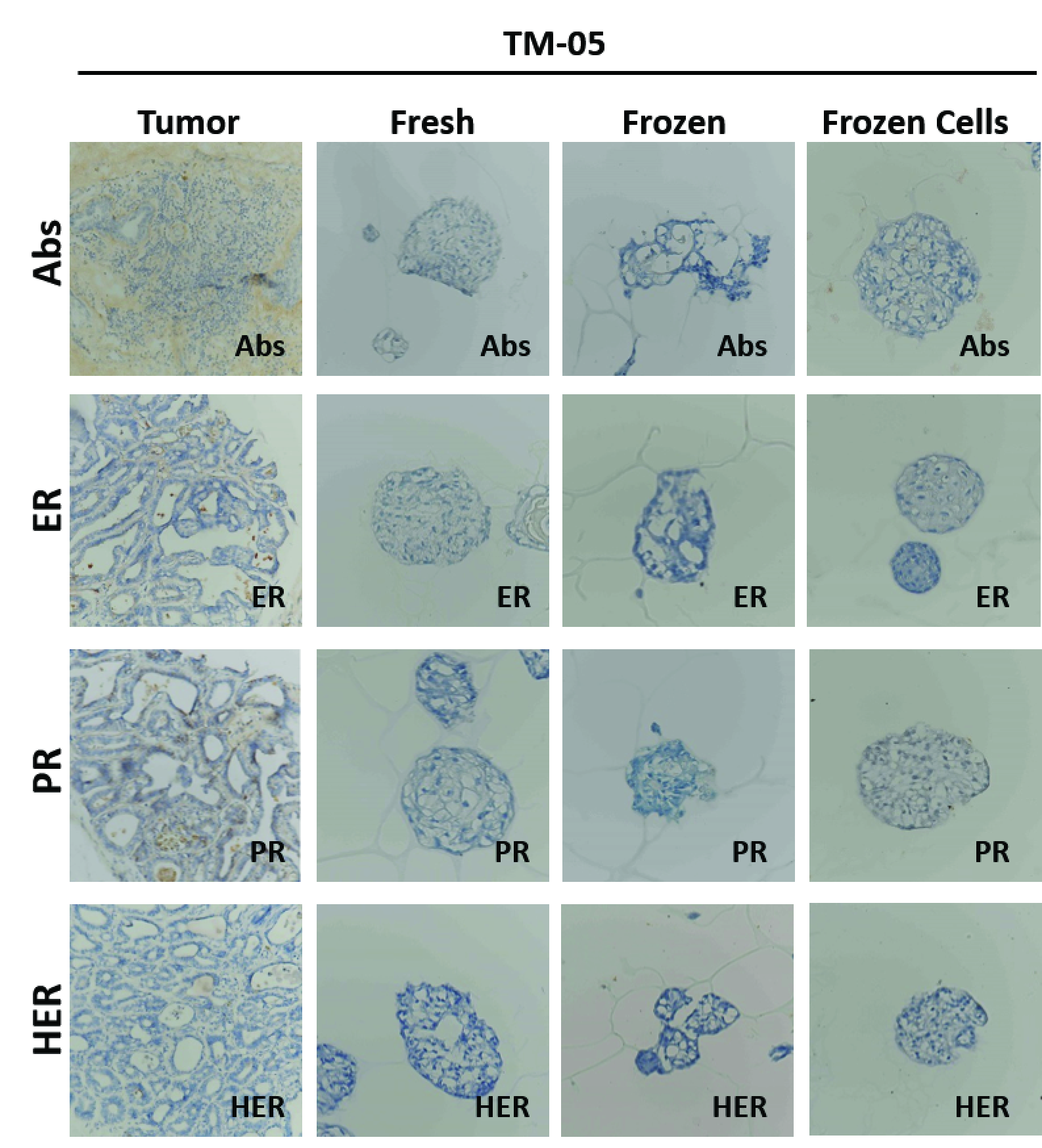

Supplement: Supplementary file 5 — Additional file 5: Fig. S3. Histology and receptor status (ER, PR, HER2) of breast cancer tumoroids. Comparative histological and immunohistochemical images of breast cancer tumoroids and their original breast cancer tissues. [file 12915_2023_1516_MOESM5_ESM.tif]
